# High-rate dead-time corrections in a general purpose digital pulse processing system

**DOI:** 10.1107/S1600577515013776

**Published:** 2015-08-07

**Authors:** Leonardo Abbene, Gaetano Gerardi

**Affiliations:** a Dipartimento di Fisica e Chimica, University of Palermo, Viale delle Scienze, Edificio 18, Palermo 90128, Italy

**Keywords:** dead-time, cascade of dead-times, time interval distribution, digital pulse processing

## Abstract

Dead-time losses are well recognized and studied drawbacks in counting and spectroscopic systems. In this work the abilities on dead-time correction of a real-time digital pulse processing (DPP) system for high-rate high-resolution radiation measurements are presented. The DPP system, through a fast and slow analysis of the output waveform from radiation detectors, is able to perform multi-parameter analysis (arrival time, pulse width, pulse height, pulse shape, *etc*.) at high input counting rates (ICRs), allowing accurate counting loss corrections even for variable or transient radiations. The fast analysis is used to obtain both the ICR and energy spectra with high throughput, while the slow analysis is used to obtain high-resolution energy spectra. A complete characterization of the counting capabilities, through both theoretical and experimental approaches, was performed. The dead-time modeling, the throughput curves, the experimental time-interval distributions (TIDs) and the counting uncertainty of the recorded events of both the fast and the slow channels, measured with a planar CdTe (cadmium telluride) detector, will be presented. The throughput formula of a series of two types of dead-times is also derived. The results of dead-time corrections, performed through different methods, will be reported and discussed, pointing out the error on ICR estimation and the simplicity of the procedure. Accurate ICR estimations (nonlinearity < 0.5%) were performed by using the time widths and the TIDs (using 10 ns time bin width) of the detected pulses up to 2.2 Mcps. The digital system allows, after a simple parameter setting, different and sophisticated procedures for dead-time correction, traditionally implemented in complex/dedicated systems and time-consuming set-ups.

## Abstract 2

The abilities on dead-time correction of a real-time digital pulse processing (DPP) system for high-rate high-resolution radiation measurements are presented. The DPP system, through a fast and slow analysis of the output waveform from radiation detectors, is able to perform an accurate estimation of the true input counting rate (ICR), a fine pulse height (energy) and shape (peaking time) analysis even at high ICRs.

## 1. Introduction   

Quantitative analysis in X-ray and γ-ray experiments requires accurate and precise estimation of input photon counting rate (ICR or ρ) and photon energy, even at high counting rate conditions. High ICR environments are typical of synchrotron applications, medical X-ray imaging, industrial imaging and security screening, and instrumentation with good counting and energy-resolving capabilities (ERPC: energy-resolved photon counting systems) is considered desirable (Fredenberg *et al.*, 2010 ▸; Kraft *et al.*, 2009 ▸; Iwanczyk *et al.*, 2009 ▸; Szeles *et al.*, 2008 ▸; Taguchi & Iwanczyk, 2013 ▸). At high ICRs, counting distortions, degradation of energy resolution and changes in energy calibration start to appear. Dead-time losses, pile-up (tail and peak pile-up) and baseline shifts (mainly due to thermal drifts, poor pole-zero cancellation and AC couplings) are the major drawbacks at high ICR environments and, therefore, high-performance spectrometers must be characterized by a well defined dead-time modeling, pile-up rejection (PUR) and baseline restoration (BLR) (Gilmore, 2008 ▸; Knoll, 2000 ▸; ICRU, 1994 ▸; Laundy & Collins, 2003 ▸).

Concerning the counting process, the dead-time (DT or τ) of the systems is the major drawback, producing both count losses and distortions of the counting statistics. Generally, when the arrival of events is random in time (*e.g.* for X-rays from tubes, from radioactive decays and from synchrotron sources with a flat fill time structure) (Bateman, 2000 ▸), dead-times are classified into two main categories: (i) non-paralyzable dead-time (also known as non-extendable, non-cumulative or type I) (Yu & Fessler, 2000 ▸) and (ii) paralyzable dead-time (also known as extendable, cumulative or type II) (Yu & Fessler, 2000 ▸). Non-paralyzable dead-time is produced at each time an event is recorded and any arrival event from the recorded time to the τ period will not be recorded. In the paralyzable model, each arrival event, whether recorded or not, produces a dead-time τ and any new arrival event with a delay less than τ from the previous arrival event extends the dead-time and will not be recorded. This model results in paralysis, *i.e.* an increasing ICR will result in a lower measured output counting rate (OCR or *R*). A third model (also known as type III) (Yu & Fessler, 2000 ▸) can be defined when a PUR is used (this technique is generally used to mitigate pile-up distortions in radiation measurements). The model of the dead-time of type III is similar to that of type II but the onset of paralysis is ‘twice as fast’, since if two events arrive within τ of each other neither event will be recorded. The transmission/throughput functions (*i.e.* the relation among OCR, ICR and DT) of these dead-times have been studied and widely presented in the literature (Arkani *et al.*, 2013 ▸; DeLotto *et al.*, 1964 ▸; Carloni *et al.*, 1970 ▸; Pommé *et al.*, 1999 ▸, Pommé, 1999 ▸; Yu & Fessler, 2000 ▸; Bateman, 2000 ▸).

Dead-time also affects the counting statistics, even if the original process can be described by a simple Poisson distribution. As shown well in both theoretical (Choi, 2009 ▸; Muller, 1967 ▸, 1971 ▸, 1972 ▸; Pommé, 1999 ▸, 2008 ▸) and experimental (Arkani & Raisali, 2015 ▸; Denecke & de Jonge, 1998 ▸; Hashimoto *et al.*, 1996 ▸; Pommé *et al.*, 1999 ▸) works, the recorded counts of a counting system with dead-time can be characterized by a non-Poisson counting uncertainty and by time-interval distributions (TIDs) different from the typical exponential shape.

Of course, dead-time distortions strongly depend on the counting rate conditions, generally related to the ρτ product, and small values are considered desirable (ρτ << 1). Therefore, at high ICRs, the counting systems should be characterized by small dead-time values to minimize the distortions and simplify the corrections.

Generally, to control the length and type of dead-time, a well defined dead-time is imposed on every event counted. Dead-time of type I or type II, greater than the dead-time of the counting chain, is typically imposed on the recorded counts. However, this approach fails at high ICRs, first, because long dead-times strongly reduce the throughput of the system and, second, it does not take into account additional counting losses due to pile-up. The presence of pile-up requires a more complex analysis of the dead-time losses, often modeled as the series arrangement of two dead-times (Choi, 2009 ▸; DeLotto *et al.*, 1964 ▸; Muller, 1972 ▸; Pommé, 2008 ▸).

Dead-time corrections can be divided into two main categories (Pommé, 2008 ▸; Michotte & Nonis, 2009 ▸; Redus *et al.*, 2008 ▸): (i) the live-time mode and (ii) the real-time mode. Live-time correction is hardware implemented. Live-time is incremented by counting a timed pulse train of known frequency only in the time intervals when the system is free to record the events. The real-time mode, off-line software implemented, is based on the knowledge of the throughput formula and the dead-time value (*i.e.* by applying the inversion of the throughput formula).

Differential methods for spectral counting correction (classified as live-time modes) have been proposed and used to also investigate variable and transient radiations (loss-free counting and zero dead-time methods) (Westphal, 2008 ▸; Upp *et al.*, 2001 ▸). These methods are based on the concept of adding *N* counts, rather than simply a single count, to a pulse height channel whenever an event was stored (*N* should equal 1 plus a weighting factor representing the estimated number of events that were lost since the last event was stored).

In this work, we will present the abilities on dead-time correction, investigated through both theoretical and experimental approaches, of a real-time digital pulse processing (DPP) system, recently developed by our group, for high-rate high-resolution radiation measurements. Currently, several spectroscopic systems are developed by using DPP techniques (Arkani *et al.*, 2014 ▸; Arnold *et al.*, 2006 ▸; Bolić & Drndarević, 2002 ▸; Cardoso *et al.*, 2004 ▸; Gerardi *et al.*, 2007 ▸; Dambacher *et al.*, 2011 ▸; Meyer *et al.*, 2001 ▸; Nakhostin & Veeramani, 2012 ▸; Papp & Maxwell, 2010 ▸), where the detector output signals, *i.e.* the output signals from charge-sensitive preamplifiers (CSPs), are directly fed into fast digitizers and then processed by using digital algorithms. As widely recognized, the digital approach gives many benefits against the analog one, among which: (i) the possibility to implement custom filters and procedures, which are challenging to realise in the analog approach, (ii) stability and reproducibility (insensitivity to pick-up noise as soon as the signals are digitized) and (iii) the possibility to perform multi-parameter analysis for detector performance enhancements and new applications. Concerning the dead-time, DPP systems are free from the dead-time due to the A/D conversion and data storage time of the traditional multichannel analyzers (MCAs). Moreover, by employing parallel or pipelined procedures, treatment dead-time (the dead-time that can arise when the on-line algorithms are applied to treat the incoming data) can be eliminated. Generally, dead-time in DPP systems is mainly due to the digital pulse shaping, allowing simple dead-time modeling and the possibility to obtain low ρτ values even at high ICRs.

Our system, based on an innovative processing architecture, is able to perform an accurate estimation of the true ICR, a fine pulse height (energy) and shape (peaking time) analysis even at high ICRs. Through two pipelined shaping branches (fast and slow channels), the system is able to minimize and correct the typical high rate distortions (dead-time distortions, pile-up and baseline shifts) in radiation measurements and, due to the pipelined analysis, no treatment dead-time is introduced. Generally, the fast channel is used to obtain the ICR and energy spectra with high throughput, while the slow channel is used to obtain energy spectra with high energy resolution. The event/pulse data from both channels (arrival time, pulse height, pulse width, peaking time), provided in listing mode, together with some housekeeping data (the starting time of the packed data acquisition, the sum of the time widths of the fast shaped pulses, the number of both fast and slow detected pulses, *etc*.), allow the correction of transmission dead-time and counting loss corrections even for variable or transient radiations.

The dead-time modeling, the throughput curves, the experimental TIDs and the counting uncertainty of the recorded events of both the fast and the slow channels, measured with a planar CdTe (cadmium telluride) detector, will be presented. The results of dead-time corrections, performed by different methods, will be also reported and discussed, pointing out the error on ICR estimation, the simplicity of the procedure and the easy implementation in a real-time mode.

The counting capabilities together with the pulse shape and height abilities, presented in our previous works (Abbene *et al.*, 2013*a* ▸,*b* ▸; Gerardi & Abbene, 2014 ▸), will give a complete overview of our digital strategy on the development of high-rate high-resolution radiation systems.

## 2. DPP system   

In this section, we will present a brief description of our DPP system. A detailed description of the system is reported in our previous work (Gerardi *et al.*, 2014). The DPP system consists of a digitizer and a PC, where the user can control all digitizing functions, the acquisition and the analysis. The pulse processing analysis is performed by using a custom DPP firmware, developed by our group and uploaded to the digitizer. We used a commercial digitizer (DT5724, CAEN SpA, Italy) (http://www.caentechnologies.com), housing four high-speed ADCs (16-bit, 100 MS/s), four buffers of external memory (8 MByte wide each) and four channel FPGAs (ALTERA Cyclone EP1C20). Each ADC channel, AC coupled, is characterized by three full-scale ranges (±1.125 V, ±0.5625 V and ±0.2813 V). The digital pulse processing is carried out by the channel FPGAs, in which our DPP method is implemented (DPP firmware). Each channel FPGA packs output data and sends them to another FPGA (ROC FPGA) that collects asynchronously the packets from all four channels and transmits them, *via* USB channel (or *via* optical link), to the PC. The PC runs a C++ program able to control all digitizer functions, to acquire packed data, to produce on-line histograms, counting rate display and to store all received information in dedicated binary files.

By using a common external clock, *N* digitizers can be assembled and synchronized to realise a digitizing system with 4×*N* channels. The acquisition start of each unit can be synchronized using a daisy-chain cascade, with the starting pulse coming from the master unit. In this way, the timing of each unit can use the same time base and starts from zero synchronously.

**Figure 1:**
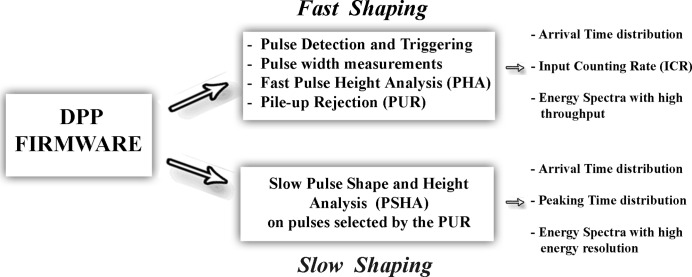
The main operations and outputs of the digital pulse processing (DPP) firmware.

**Figure 2:**
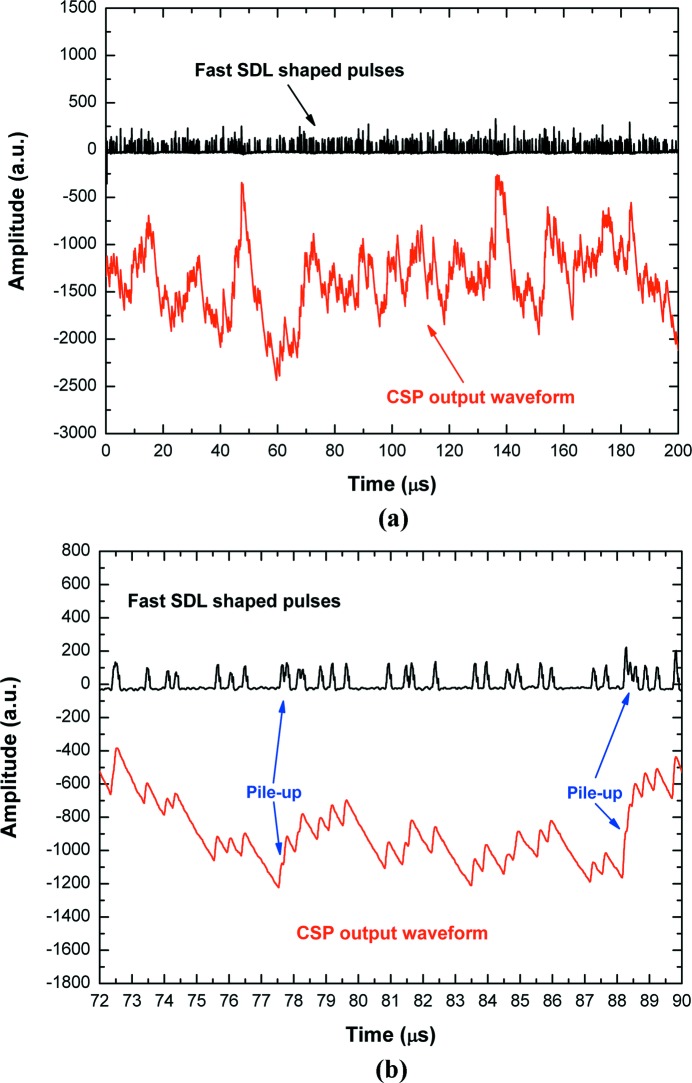
(*a*) The digitized waveform from the preamplifier (CSP output waveform) and the pulses from the fast SDL shaping. (*b*) A zoom of the signals clearly shows the fast detection of the pulses from the waveform; some piled-up pulses are also shown. The pulses represent X-rays from an Ag-target X-ray tube impinging on a semiconductor detector (CdTe detector) with an ICR of 2.2 Mcps.

The DPP firmware was developed by our group and successfully used for both off-line and on-line analysis (Abbene *et al.*, 2010 ▸, 2012 ▸, 2013*a* ▸,*b* ▸, 2015 ▸; Abbene & Gerardi, 2011 ▸; Gerardi & Abbene, 2014 ▸). The DPP method is able to perform multi-parameter analysis (event arrival time, pulse shape, pulse height, pulse time width, *etc*.) even at high ICRs. A general overview of the method is presented below (see also Fig. 1 ▸). The DPP method is based on two pipelined shaping steps: a *fast* and a *slow* shaping. The preamplifier output waveform (CSP output waveform) is shaped by using the classical single delay line (SDL) shaping technique (Knoll, 2000 ▸). SDL shaping is obtained by subtracting from the original pulse its delayed (by using a programmable delay time) and attenuated fraction. SDL shaping gives short rectangular output pulses with fast rise and fall times. In fact, the falling edge of the pulse is a delayed mirror image of the leading edge. These features make SDL shaping very appealing for timing and pulse shape and height analysis (PSHA) at both low and high counting rates. Through the fast SDL shaping the following operations are performed: (i) pulse detection and time-tag triggering, (ii) time width measurement of the SDL-shaped pulses, (iii) fast pulse height analysis (PHA), that provides energy spectra with high throughput, and (iv) pile-up rejection for the slow branch. Concerning the pulse detection, the trigger is generated and time-stamped through the ARC (amplitude and rise time compensation) timing marker (at the leading edge of the SDL pulses) and its amplitude (25% of the peak value) defines the new amplitude threshold (ARC threshold) for SDL pulse width estimation. The estimation of the ARC cross timing is improved by using a linear interpolation (time resolution < 1 ns). The width of each SDL pulse is calculated from the difference between the times when the leading and the falling edges cross the ARC threshold. Through the fast branch, the system is able to provide, for each detected event, the following results: (i) trigger time stamp, (ii) pulse width, (iii) fast pulse height. Fig. 2 ▸ shows the CSP output waveform and the fast shaped pulses, related to X-rays from an Ag-target X-ray tube impinging on a CdTe detector with an ICR of 2.2 Mcps (cps = counts s^−1^) (the experimental set-up is described in §4).

**Figure 3:**
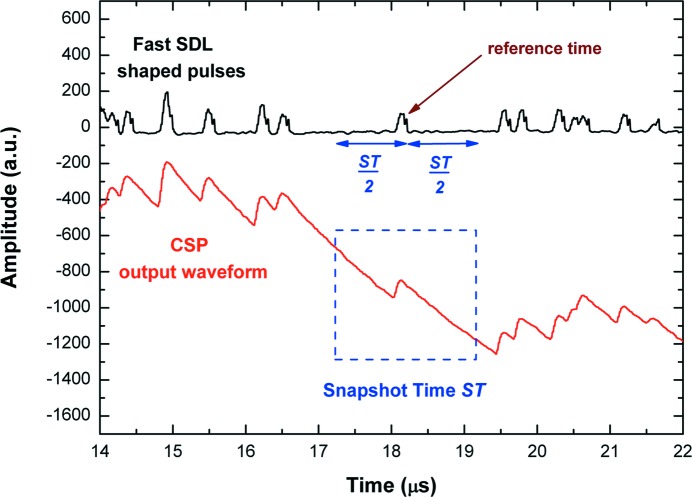
Selection, through the PUR, of a time window of the CSP waveform for the slow shaping. Each selected time window is termed ‘Snapshot’, while the width of this window is termed ‘Snapshot Time’ (ST). The selection is related to the reference time of each fast SDL pulse: a pulse is accepted if it is not preceded and not followed by another pulse in the ST/2 time window periods; if two detected fast SDL pulses are within ST/2 of each other, then neither pulse will be selected. The PSHA is performed on the snapshot window with benefits at high ICRs (minimization of baseline shifts, *etc*.). The pulses represent X-rays from an Ag-target X-ray tube impinging on a semiconductor detector (CdTe detector) with an ICR of 2.2 Mcps. A ST of 2 µs was used.

The PUR performs a selection of time windows of the CSP waveform for the slow shaping (Fig. 3 ▸). Each selected time window of the CSP waveform is termed ‘Snapshot’, while the width of this window, user-chosen, is termed ‘Snapshot Time’ (ST). The selection is related to the reference time of each fast SDL pulse (it occurs near the maximum amplitude of the related CSP pulse), *i.e.* to the time when the falling edge of the SDL pulse crosses the ARC threshold. If two detected fast SDL pulses are within ST/2 of each other, then neither pulse will be selected; *i.e.* a pulse is accepted if it is not preceded and not followed by another pulse in the ST/2 time window periods. We stress that the PUR only works on the temporal positions of the CSP pulse peaks, *i.e.* it selects the snapshots before any useful operation for slow shaping. The slow shaping is characterized by two main features: (i) it performs the PSHA on each selected snapshot, and (ii) due to an automatic baseline restoration (based on the analysis on single pulses), it allows high rate measurements. The pulse height analysis (that provides energy spectra with high energy resolution of each PUR selected event) is performed by applying an optimized low-pass filter (*e.g.* trapezoidal filter) to all the samples of each slow SDL-shaped pulse. The energy resolution strongly depends on the ST values; as the shaping time of classic analog systems, long ST values give better energy resolution. Through the slow branch, the system is able to provide, for each selected pulse, the following results: (i) trigger time stamp, (ii) pulse height and (iii) the peaking time. The shape (peaking time) of the pulses and its correlation with the pulse height is very helpful for improving the detector performance. Pulse shape discrimination (PSD) techniques were successfully used, in our previous works (Abbene & Gerardi, 2011 ▸; Abbene *et al.*, 2012 ▸, 2013*a* ▸,*b* ▸, 2015 ▸; Gerardi & Abbene, 2014 ▸), to minimize incomplete charge collection effects, pile-up and charge sharing.

We stress that this PSHA, performed on isolated time windows containing a single CSP pulse, allows a strong reduction of the corruptions that the traditional analysis produces to adjacent pulses (residual tails at the end of shaped pulses), thus minimizing baseline shifts at high ICRs.

The output results from both channels are provided in listing mode, where each list is characterized by a user-chosen number of event-sequences (typical fast channel sequence: arrival time, fast energy and pulse width; typical slow channel sequence: arrival time, slow energy and peaking time). Moreover, to perform investigations, with high time resolution, on variable and transient radiations (multiscaling and spectral modes), each data list is tied to some housekeeping data, such as: the starting time of the packed data acquisition, the sum of the time widths of the detected pulses (total detection dead-time), total number of fast shaped pulses, total number of analysed events (after PUR), total number of pile-up events, *etc*. These data, continuously updated, allow the analysis of the time evolution of the total photon counting rate to be performed and allow the detection and the measurement of any transmission dead-time. Of course, the time resolution of this analysis depends on the counting rate and the chosen number of packed event-sequences. Moreover, the data within each list (*i.e.* the sequences: arrival time, energy, *etc*.) allow a finer analysis of the time evolution of the energy spectra (*e.g.* changes of the rate of some energy lines in the spectrum) and loss-counting corrections can be easily performed.

## 3. Dead-time modeling   

In this section, the dead-time modeling, throughput functions, time-interval distributions and counting uncertainties of the two shaping channels will be presented and discussed.

### 3.1. Dead-time of the fast channel   

As will be shown in the experimental results (§5), the dead-time of the fast channel can be modeled as a single paralyzable dead-time (type II). The pulse detection is performed in the fast channel by looking for the fast SDL output pulses exceeding an amplitude threshold (leading edge detection). Pulses that are large enough to cross this threshold are counted. The width of the fast SDL output pulses at the threshold causes an extending dead-time (type II). If a second pulse arrives while the first pulse is still above the threshold, the second pulse overlays the first, and extends the dead-time by its width from its arrival time. Because the system counts threshold crossings, it will count only the first pulse. If *τ*_F_ is the fast dead-time, *R*_F_ the output counting rate and ρ the input counting rate, the throughput function is given by the following relation (Gilmore, 2008 ▸; Knoll, 2000 ▸),
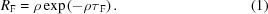
As is widely reported in classic textbooks on radiation detection (Gilmore, 2008 ▸; Knoll, 2000 ▸), equation (1) is obtained by calculating the probability of time-intervals, between consecutive events, longer than τ_F_, *i.e.* by integrating the exponential time-interval distribution, typical of a Poisson process, between τ_F_ and ∞. As discussed in the *Introduction* [Sec sec1], dead-time also affects the shape of the TID. The TID of the recorded events after a single dead-time of type II can be described by the following function (Pommé *et al.*, 1999 ▸; Pommé, 2008 ▸; Muller, 1971 ▸),
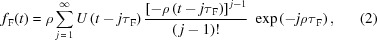
where 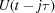 is the Heaviside step function. Due to the effect of the dead-time, the TID, described by (2), is represented by a piecewise polynomial function, *i.e.* characterized by a different shape from the exponential function, typical of a Poisson process.

**Figure 4:**
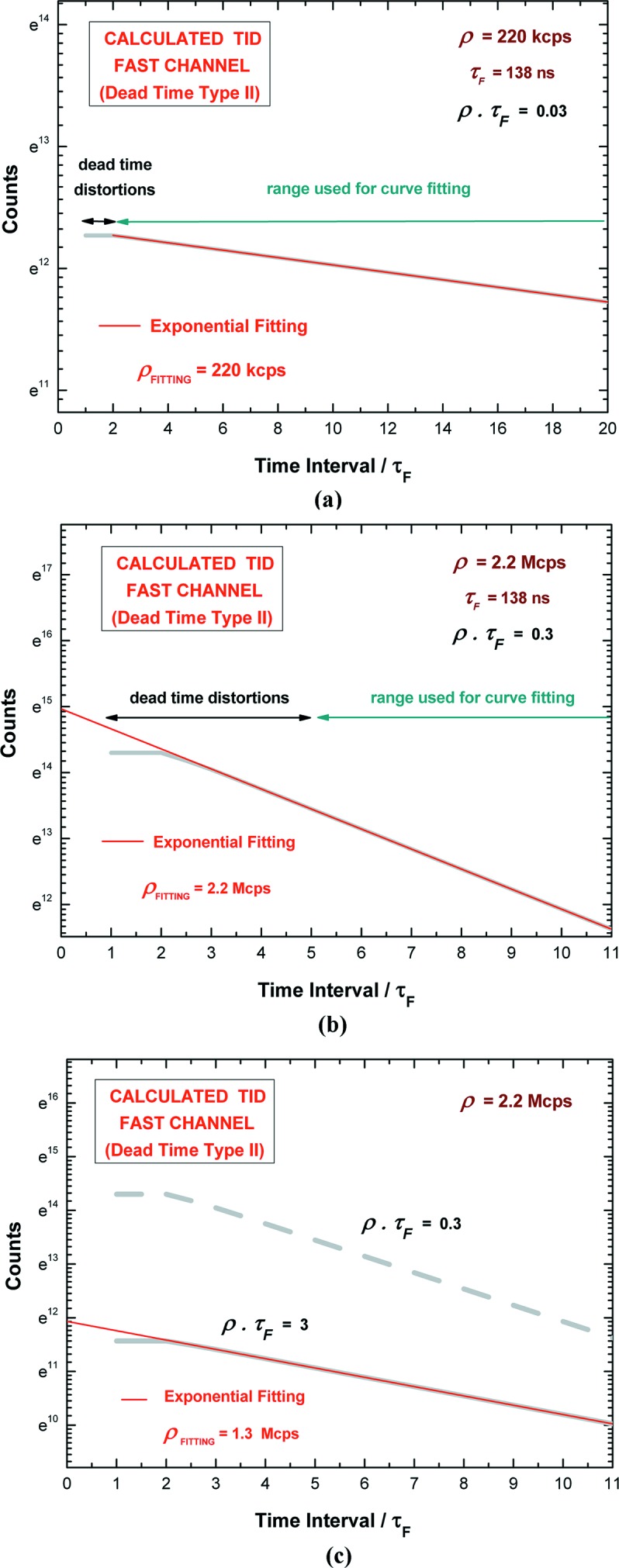
Calculated time-interval distributions (TIDs) of the recorded counts of the fast channel (dead-time of type II) by using equation (2) at different ρτ_F_ values. (*a*) TID (thick gray line) at 220 kcps (ρτ_F_ = 0.03); there are no dead-time distortions at time intervals longer than 2τ_F_ and the exponential best fitting (thin red line), performed at times >2τ_F_, gives an estimated ρ_FITTING_ equal to the true ρ. (*b*) TID (thick gray line) at 2.2 Mcps (ρτ_F_ = 0.3); here, there are dead-time distortions at time intervals smaller than 5τ_F_; the exponential best fitting (thin red line), at time intervals >5τ_F_, gives an estimated ρ_FITTING_ equal to the true ρ. (*c*) TIDs at ρ = 2.2 Mcps; the TID at ρτ_F_ = 0.3 (dashed gray line, τ_F_ = 138 ns) is compared with the TID at ρτ_F_ = 3 (solid gray line, τ_F_ = 1.38 us); the exponential fitting of the TID at ρτ_F_ = 3 gives an estimated ρ_FITTING_ = 1.3 Mcps (different from the true ρ = 2.2 Mcps), even at time intervals greater than 20τ_F_.

However, at low ρτ_F_ product values and at long time intervals (if compared with the involved dead-time), the TID tends towards the Poisson exponential shape. In the following, we will present some calculated TIDs (Fig. 4 ▸) of a single dead-time of type II [by using equation (2)] and we will discuss the limits of the exponential approximation of the TID. By using equation (2), we calculated the TIDs at three different ρτ_F_ product values of 0.03, 0.3 and 3. The ρτ_F_ values are related to the experimental conditions presented in this work: ρ values from 220 kcps to 2.2 Mcps and a dead-time τ_F_ equal to 138 ns (the estimation of the fast dead-time τ_F_ will be presented in §5). Fig. 4(*a*) ▸ shows the calculated TID by using ρ = 220 kcps (ρτ_F_ = 0.03). The TID is zero between 0 and τ_F_, constant between τ_F_ and 2τ_F_, and at time intervals longer than 2τ_F_ can be modeled with an exponential function. Indeed, by performing an exponential fitting, at times >2τ_F_, we obtained ρ_FITTING_ = 220 kcps, that is equal to the ρ used for the calculus of the TID. Fig. 4(*b*) ▸ shows the TID at 2.2 Mcps (ρτ_F_ = 0.3). Here, the TID follows an exponential shape at times longer than 5τ_F_. Fig. 4(*c*) ▸ shows two TIDs at 2.2 Mcps but characterized by different ρτ_F_ product values: ρτ_F_ = 0.3 (dashed gray line, with τ_F_ equal to 138 ns) and ρτ_F_ = 3 (solid gray line, with τ_F_ equal to 1.38 µs). The different slope of the two TIDs is clearly evident. In particular, the exponential fitting of the TID with ρτ_F_ = 3 gives ρ_FITTING_ = 1.3 Mcps, even at time intervals > 20τ_F_. Therefore, at high ρτ_F_ product values, the exponential fitting, despite the good agreement with the calculated TID data, gives an error on the ρ estimation.

Since the dead-time models of type I and II lead to identical results in the limit of small dead-time losses (*i.e.* small ρτ values), the exponential modeling of the TID of the fast shaped pulses can be compared with the similar behaviour of the non-paralyzable (type I) dead-time, which is characterized by zero value for *t* ≤ τ_F_ and by an exponential TID for *t* > τ_F_ (Muller, 1967 ▸; Pommé, 1999 ▸).

These results justify the following exponential modeling of the TID of the fast shaped pulses for small ρτ_F_ values. At small ρτ_F_ values (ρτ_F_ ≤ 0.03), we will use the following piecewise model for the TID of a single dead-time τ_F_ of type II (fast channel),
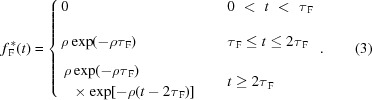
The counting uncertainty of the recorded counts are also affected by dead-time. In particular, the relative uncertainty on the recorded counts *N*_F_ of the fast channel (type II) is given by the following relation (Pommé *et al.*, 1999 ▸; Yu & Fessler, 2000 ▸),
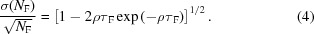



### 3.2. Dead-time of the slow channel   

The slow channel performs a multi-parameter analysis (arrival time, energy and peaking time) on each pulse selected by the PUR, within a time window of the CSP waveform equal to ±ST/2, centered at the peak position. The value of this window, chosen by the user, generally represents the best compromise between the energy resolution and the throughput in the slow energy spectra (the ST acts as the shaping time constant of an analog shaping amplifier). The dead-time of the slow channel should be modeled as a single dead-time of type III, *i.e.* characterized by the following throughput function (Yu & Fessler, 2000 ▸),
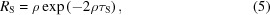
where *R*_S_ is the output counting rate and τ_S_ is the slow dead-time equal to ST/2. However, due to the finite width of the pulses of the fast channel, higher throughputs than the values expected from equation (5) would be observed (*i.e.* a lower dead-time than ST/2) (Pommé *et al.*, 1999 ▸, 2008 ▸; Michotte & Nonis, 2009 ▸; Yu & Fessler, 2000 ▸). Indeed, the slow channel should be modeled through the cascade of two paralyzable dead-times: the first of type II (fast dead-time equal to τ_F_) and the second of type III (slow dead-time τ_S_ equal to ST/2).

In the following, a simple modeling of the cascade of type II (with ρτ_F_ << 1) and type III will be presented. To our knowledge, the modeling of the cascade of dead-times of type II with type III has not been presented in the literature. For an exponential TID, the probability that an event can be preceded or followed by another event, within the time interval τ_S_, is the same. We define *P*_LOSS_ as the probability that one pulse is rejected by the arriving of a new event, within the interval (0, τ_S_). The probability that one event is accepted by our PUR, *i.e.* no event is present in the two time intervals (−τ_S_, 0) and (0, τ_S_), is equal to (1 − *P*_LOSS_)(1 − *P*_LOSS_). At low ρτ_F_ product values (ρτ_F_ << 1), *P*_LOSS_, by using the TID of equation (3), is given by 
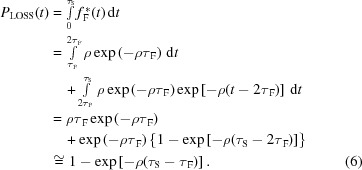
Therefore, the output counting rate 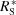 of the slow channel can be given by
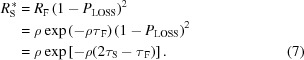
Notice that the same result is obtained, without approximation, if the fast dead-time is of type I.

The relative uncertainty on the recorded counts *N*_S_ of the slow channel can be written as (Pommé *et al.*, 1999 ▸; Yu & Fessler, 2000 ▸)
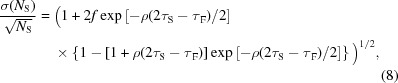
where *f* is a fraction of the counts in the slow energy spectrum. Equation (8) is derived by the theoretical relation for a single dead-time of type III; we take into account the cascade of dead-time of type II and dead-time of type III by using the cascade corrected total dead-time (*i.e.* 2τ_S_ − τ_F_).

## 4. Experimental procedures   

To investigate the counting capabilities of the DPP system, a planar CdTe detector was used (XR100T-CdTe, S/N 6012, Amptek, USA) (http://www.amptek.com), with a thickness of 1 mm (absolute efficiency of 64% at 100 keV) and equipped with a resistive-feedback CSP (decay time constant of the resistive-feedback circuit is around 100 µs). The gain of the CSP is 0.82 mV keV^−1^ and the rise time of the CSP output pulses is around 60 ns (59.5 keV X-rays). As is well known, CdTe/CdZnTe detectors (1–2 mm thick) are very appealing for X-ray spectroscopy in the energy range 1–100 keV (Auricchio *et al.*, 2011 ▸; Del Sordo *et al.*, 2009 ▸; Owens, 2006 ▸; Takahashi & Watanabe, 2001 ▸; Turturici *et al.*, 2014 ▸, 2015 ▸).

The high-rate spectroscopic abilities of the DPP system, connected to the CdTe detector, were investigated in our previous works (Abbene *et al.*, 2013*a* ▸,*b* ▸; Gerardi & Abbene, 2014 ▸). Table 1 ▸ shows the spectroscopic response of the system at low and high rates, in terms of energy resolution (FWHM), at 59.5 keV (^241^Am source). The electronic noise of the CdTe detector coupled to the DPP system (by using ST = 30 µs) is 0.4 keV (FWHM). The results highlight, beside the excellent high-rate spectroscopic abilities, the flexibility of the system to perform measurements for both optimum energy resolution or high throughput.

In this work, we measured the response of the system to an Ag-target X-ray tube (Amptek, Inc. USA) with Al (1 mm thick) and Ag (25.4 µm thick) filters. X-ray spectra were measured by using a tube voltage of 30 kV and tube current values between 5 µA and 60 µA (ICR up to 2.2 Mcps).

## 5. Measurements and results   

In this section, experimental results on the counting rate capabilities of the system, through the fast and slow channels, are shown. As will be presented in the following subsections, the DPP system is characterized by two main features: (i) the dead-time modeling of both the fast and the slow channel is well defined and (ii) thanks to the low dead-time values of the fast channel, accurate estimation of the true ICR can be performed.

### 5.1. Dead-time correction and counting rates in the fast channel   

**Figure 5:**
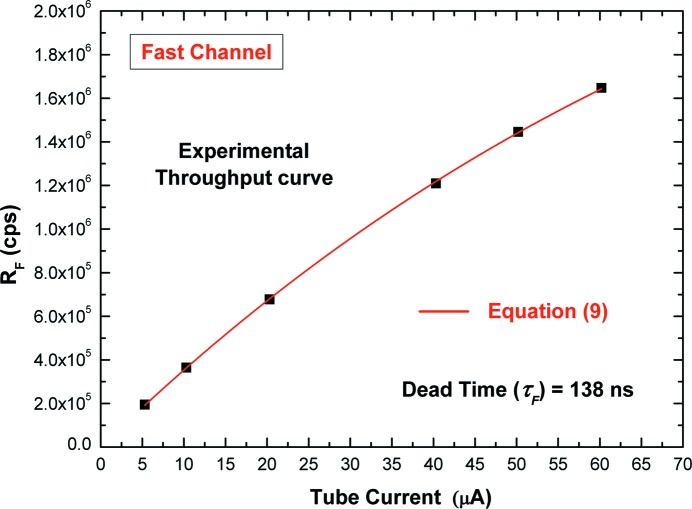
Experimental throughput curve from the fast channel. The experimental points are in good agreement with the throughput function (red line) of a single paralyzable dead-time (the coefficient of determination is equal to 0.9999; this parameter indicates how well experimental data fit the model and a value close to 1 indicates that the model perfectly fits the data) (Draper & Smith, 1998 ▸). Errors of experimental points are too small to be visible in the figure.

Fig. 5 ▸ shows the measured throughput curve (*i.e.* the *R*_F_ *versus* tube current) of the fast channel. Each experimental point was obtained by evaluating the mean value of *R*_F_ values of 400 acquisitions (each acquisition consists of 20000 events). The experimental curve is in good agreement with the typical throughput function of the single paralyzable dead-time model [the dead-time model described by equation (1)].

Through a curve fitting with the following function,
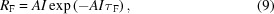
where *I* is the tube current and *A* is a constant, we estimated, with a confidence level (CL) of about 99.7%, τ_F_ = (138.0 ± 0.6) ns. Therefore, it is possible to estimate the input counting rate ρ by applying the real-time dead-time correction, *i.e.* by solving the throughput equation (1) iteratively. Of course, this method requires the experimental measurement of the throughput curve and therefore the measurement of multiple X-ray spectra at different ICRs (multiple measurements). In the following, a different method, able to perform accurate estimation of ρ with a single measurement (*i.e.* by performing a measurement at a single ICR condition), will be presented.

**Figure 6:**
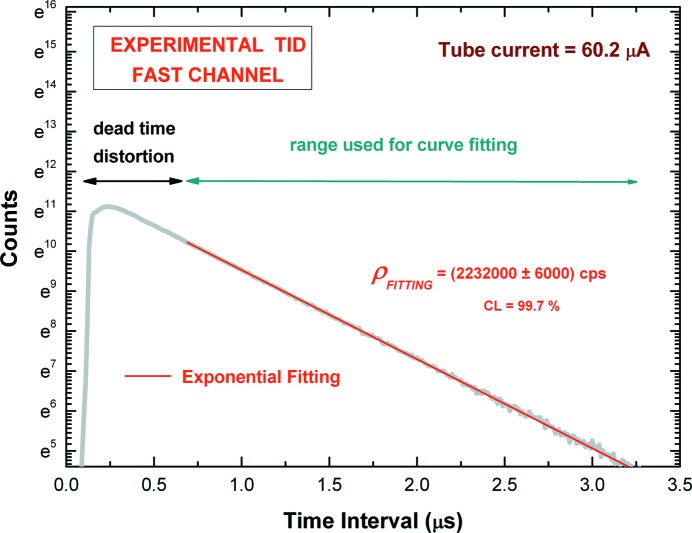
Measured time-interval distribution (TID) of the events of the fast channel (thick gray line) at 2.2 Mcps (ρτ_F_ = 0.3) with a time bin width of 10 ns. The exponential best fitting (thin red line), performed at time intervals >5τ_F_, is in good agreement with experimental data (the coefficient of determination is equal to 0.9997).

**Figure 7:**
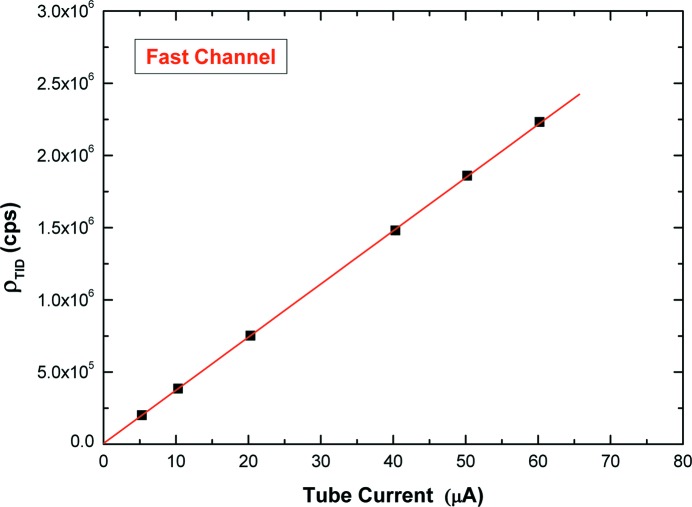
ρ_TID_ estimated from the measured time-interval distributions (TIDs) of the pulses of the fast channel *versus* the tube current (nonlinearity < 0.5%).

As discussed in §3 and reported in the literature (Arkani & Raisali, 2015 ▸; Denecke & de Jonge, 1998 ▸; Pommé *et al.*, 1999 ▸), due to the small dead-time τ_F_ of the fast channel (138 ns), the simple exponential fitting of the experimental TID, at time intervals >5τ_F_, can give an accurate estimation of the input counting rate up to 2.2 Mcps (ρτ_F_ = 0.3). Fig. 6 ▸ shows the experimental TID, through the fast channel at 2.2 Mcps; the trigger times of the event-data, histogrammed with a time bin width of 10 ns, were used. The exponential fitting, performed at time intervals >5τ_F_, gives ρ_TID_ = (2232000 ± 6000) cps (CL = 99.7%). The estimated ρ_TID_ from the measured TIDs *versus* the tube current is characterized by a very good linear behavior (nonlinearity < 0.5%), as shown in Fig. 7 ▸. Moreover, to check the ρ_TID_ values, we also estimated τ_F_ by fitting the throughput curve (*R*_F_ *versus* ρ_TID_; for simplicity, this curve was not reported in the paper) with the single paralyzable function [equation (1)], obtaining a dead-time τ_F,TID_ = (137 ± 0.9) ns (CL = 99.7%), in good agreement with the dead-time (138 ± 0.6 ns) estimated from the experimental throughput curve (*i.e.* *R*_F_ *versus* tube current).

**Figure 8:**
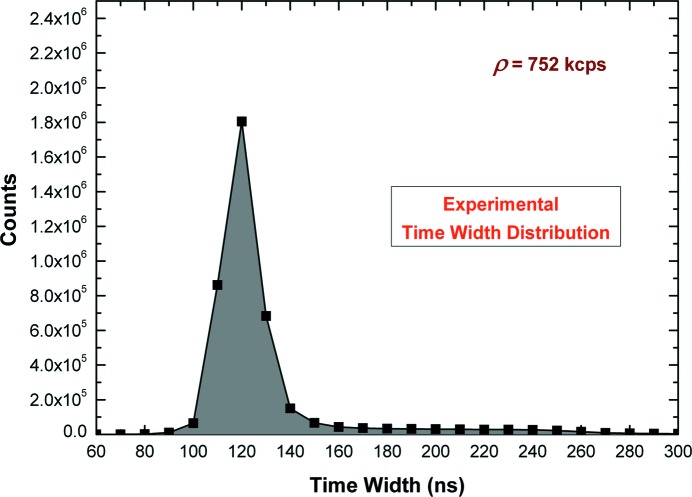
Measured time width distribution of the fast pulses at 752 kcps.

The digital system, through the fast channel, is able to perform the estimation of the true ρ by using several dead-time correction methods. In the following, we summarize all techniques used to estimate the true ρ, pointing out if each method needs a single measurement of multiple measurements:

(i) ρ_REAL_, obtained through the real-time correction [*i.e.* by using equation (1)] from the measured throughput curves (multiple measurements);

(ii) ρ_TID_, estimated from the exponential best fit of the measured TIDs (single measurement);

(iii) ρ_LIVE_, obtained through the relation *N*_F_/(*T*_acq_ − *T*_width_), where *N*_F_ is the total number of the detected pulses by the fast channel, *T*_acq_ is the total real acquisition time, while *T*_width_ is the total detection dead-time, calculated as the sum of the time widths of the fast shaped pulses (single measurement);

(iv) ρ_TW_, obtained by using a different real-time correction, based on the paralyzable throughput function [equation (1)] and the dead-time τ_FAST,TW_ estimated through the mean value of the time widths of the detected pulses (single measurement). Fig. 8 ▸ shows the time width distribution of the fast pulses at ρ = 752 kcps. From the time width data, we obtained a constant τ_FAST,TW_ = (129 ± 10) ns (CL = 99.7%) for all counting rates (from 200 kcps to 2.2 Mcps).

**Figure 9:**
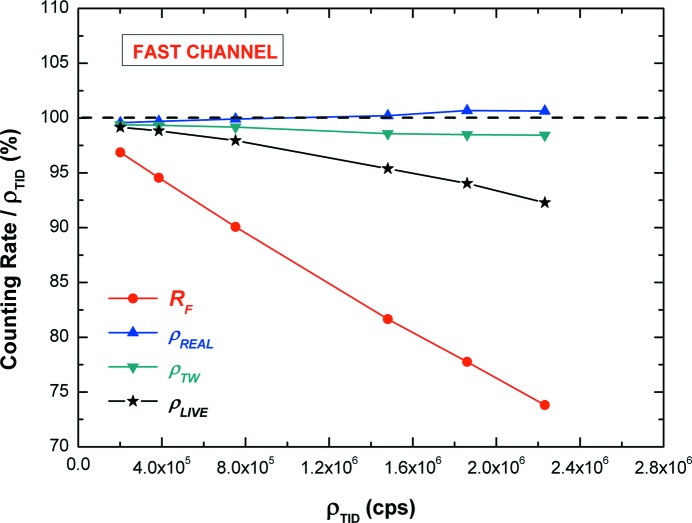
ρ values estimated through different dead-time correction methods. Each ρ value is related to the ρ_TID_. The *R*_F_ values, related to ρ_TID_, are also reported.

The ρ values (related to ρ_TID_), estimated through the various correction methods, are shown in Fig. 9 ▸. We used ρ_TID_ as the reference input counting rate, due to the good linearity with the tube current. To better point out the counting corrections, the *R*_F_ values are also reported in Fig. 9 ▸. At 200 kcps, all correction methods are characterized by low errors, <0.8%. By applying the real-time correction, up to counting rates of about 2.2 Mcps, the uncertainty of ρ_REAL_ is <0.6%, while the error on ρ_TW_ is <1.6%. For comparison purposes, the live correction was also reported. As clearly shown in Fig. 9 ▸, the error on ρ_LIVE_ (<7.8% at 2.2 Mcps) is greater than for the other correction methods, mainly due to its major sensibility to the pulse pile-up (Pommé, 2008 ▸; Michotte & Nonis, 2009 ▸); moreover, ρ_LIVE_ values are always lower than the expected values as happens in the live time correction.

**Figure 10:**
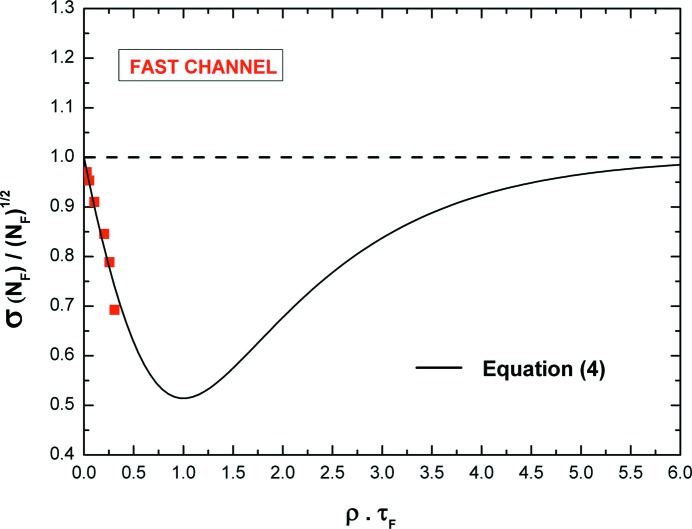
Ratio between the measured standard deviation of *N*_F_ and (*N*_F_)^1/2^ (*i.e.* the expected standard deviation in a Poisson process) *versus* the ρτ_F_ product values.

We calculated the standard deviation of the recorded counts of 400 measurements. Fig. 10 ▸ shows the ratio between the measured standard deviation of *N*_F_ and (*N*_F_)^1/2^ (*i.e.* the expected standard deviation in a Poisson process) *versus* the ρτ_F_ product values. At 2.2 Mcps (*i.e.* ρτ_F_ = 0.3), the counting uncertainty is clearly less than the value expected from Poisson statistics, with a percentage deviation of about 30%. The experimental points are in agreement with equation (4) and with the values obtained in the literature, in both simulations and experiments (Pommé *et al.*, 1999 ▸; Yu & Fessler, 2000 ▸) with counting systems characterized by a single paralyzable dead-time. This result points out that the system is able to associate the proper standard deviation on the fast recorded counts.

### 5.2. Dead-time correction and counting rates in the slow channel   

**Figure 11:**
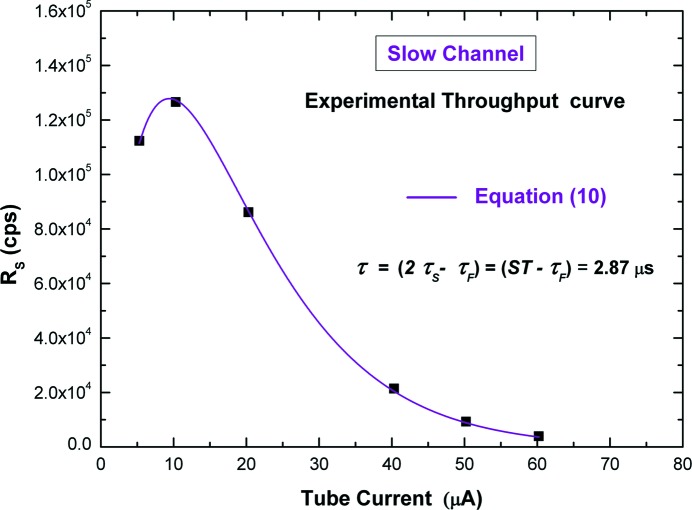
Measured throughput curve from the slow channel. The throughput function of the cascade of dead-time of type II and type III [equation (7)] is in good agreement with the experimental points (the coefficient of determination is equal to 0.9998). Errors of experimental points are too small to be visible in the figure.

Fig. 11 ▸ shows the measured throughput curve (*i.e.* *R*_S_ *versus* tube current) of the slow channel by using ST = 3 µs. Each experimental point was obtained by evaluating the mean value of *R*_S_ of 400 acquisitions (each acquisition consists of the selected events by the PUR from the 20000 events from the fast channel; the number of the selected events changes with the rate). The experimental curve was fitted with the following equation,
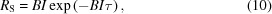
where *I* is the tube current and *B* is a constant, giving a total dead-time τ equal to (2.87 ± 0.04) µs (confidence level CL = 99.7%). This value is equal to (2τ_S_ − τ_F_), clearly pointing out the good agreement between the experimental curve and the throughput function of the cascade of type II and type III dead-times up to ρτ_F_ = 0.3 [equation (7)].

**Figure 12:**
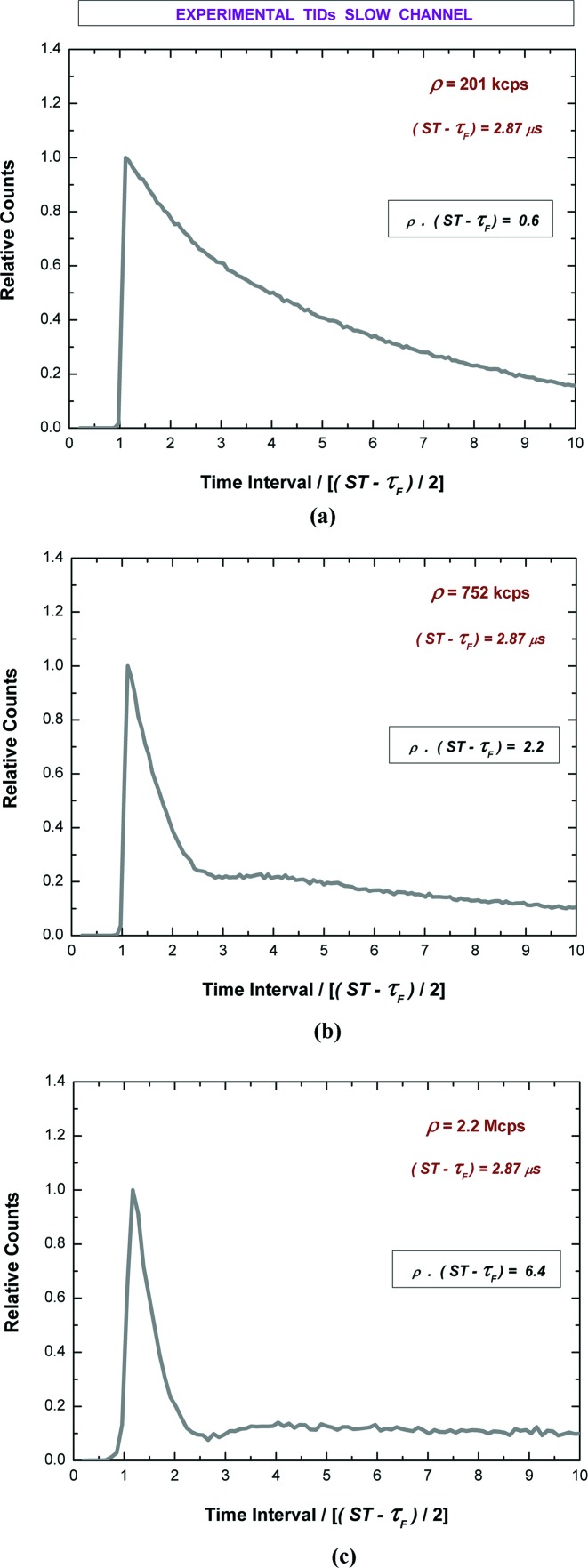
Measured time-interval distributions (TIDs) of the events of the slow channel at (*a*) 201 kcps, (*b*) 752 kcps and (*c*) 2.2 Mcps, with a time bin width of 100 ns. The counts were normalized to the maximum number of detected events.

Fig. 12 ▸ shows the measured TIDs, through the slow channel, at 200 kcps, 752 kcps and at 2.2 Mcps, with a time bin width of 100 ns. The shapes of the distributions show an agreement with both simulated and experimental TIDs (single dead-time of type III) in the literature (Pommé *et al.*, 1999 ▸). These results show that the low dead-time of the fast channel produces negligible effects in the TIDs of the slow channel. However, due to the high distortions of the slow dead-time, the measured TIDs from the slow channel do not allow accurate *ρ* estimations through a simple exponential fitting. Therefore, each slow channel high-resolution spectrum should be tied to the *ρ* information provided by the fast channel, characterized by very low dead-time distortions.

In the following, we present some appealing strategies, in terms of both simplicity and accuracy, that can be used to provide the scaling ratio for the spectral counts and its error with a single measurement:

(i) Estimation of ρ through the exponential fitting of the TID from the fast channel and calculation of the scaling ratio *K* = ρ_TID_/*R*_S_; this is the best strategy in terms of accuracy, but requiring the implementation of a best-fit procedure;

(ii) Estimation of τ_F_ through the mean value of the time width of the fast pulses and calculation of 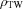 by inversion of the formula (1); the scaling ratio is given by
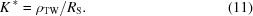



(iii) Estimation of τ_F_ through the mean value of the time width of the fast pulses; since *R*_S_ follows the relation
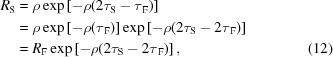
it is possible to estimate the scaling ratio *K* ^**^ through 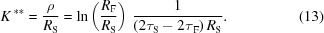
At 2.2 Mcps, by using the estimated τ_F,TW_ = (129 ± 10) ns, the measured *R*_S_, *R*_F_ and 2τ_S_ = ST = 3 µs, a ρ value (2200000 ± 50000 cps) was obtained, through equation (13), with a percentage deviation of 1.4% from the ρ_TID_ (2232000 ± 6000 cps); therefore, taking into account this maximum error, it is possible to correct the counts in the slow spectra though this simple procedure up to 2.2 Mcps.

**Figure 13:**
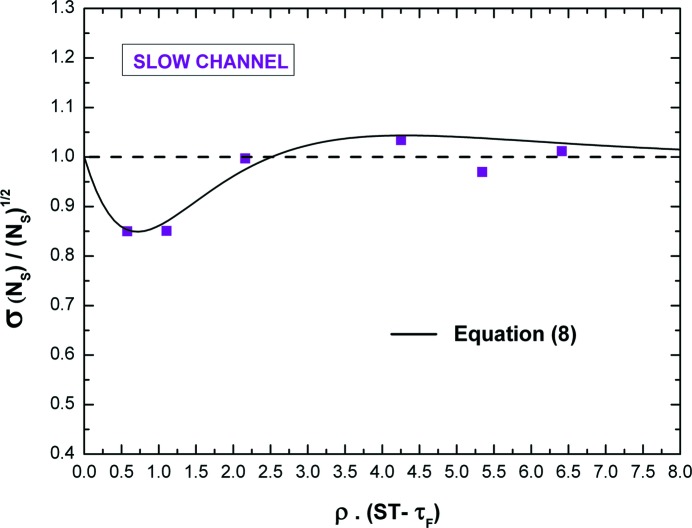
Ratio between the measured standard deviation of *N*_S_ and (*N*_S_)^1/2^ *versus* the ρ(ST − τ_F_) product values.

Fig. 13 ▸ shows the ratio between the measured standard deviation of *N*_S_ and (*N*_S_)^1/2^ *versus* the ρ(2τ_S_ − τ_F_) product values. We calculated the standard deviation of the recorded counts of 400 measurements. As in the fast channel, the counting uncertainty is different from the value expected from Poisson statistics (maximum percentage deviation of about 15%), but this difference is much smaller than the measured fast channel one. Moreover, the experimental points are in agreement with equation (8) and with the values obtained in the literature, in both simulations and experiments (Pommé *et al.*, 1999 ▸) with counting systems characterized by a single pile-up rejection (type III).

Of course, a smaller difference with the Poisson counting uncertainty can be gradually obtained when small fractions of the spectral counts (ROI) are considered (Pommé *et al.*, 1999 ▸).

## 6. Conclusions   

The high rate abilities of a real-time DPP system on dead-time correction are presented. The system through a fast and a slow channel is able to provide counting and energy spectra at different resolution and throughput conditions. The results of X-ray spectra measurements (up to 2.2 Mcps) highlight two main features of the DPP system: (i) the dead-time modeling of both the fast and the slow branch is well defined: a single dead-time of type II for the fast channel and a cascade of dead-time of type II and type III for the slow channel; and (ii) thanks to the low dead-time values of the fast channel, low dead-time distortions are present and accurate estimation of the true input counting rate can be performed. Accurate counting rate estimations were performed by using the time widths and the time-interval distributions of the pulses from the fast channel.

Moreover, the DPP output results, provided in timed packed listing mode, together with the housekeeping data, allow counting loss corrections even for variable or transient radiation sources, with time resolutions depending on the ICR and the chosen number of radiation events.

We stress that the digital system allows, after a simple parameter setting, different and sophisticated procedures for dead-time correction, traditionally implemented in complex/dedicated systems and time-consuming set-ups.

## Figures and Tables

**Table 1:** Spectroscopic response of the DPP system coupled to the CdTe detector at 59.5keV (^241^Am source)

Energy resolution FWHM (%) at 59.5keV	ICR (kcps)	Throughput (%)	Shaping mode	Set-up
1.3	0.2	99	Slow PSHA	High energy resolution (ST = 30s)
2.5	850	1.5	Slow PSHA and pulse shape discrimination (PSD)	High energy resolution (ST = 3s)
7.2	850	80	Fast PHA	High throughput (fast SDL shaping delay of 200ns)
